# PKC is an indispensable factor in promoting environmental toxin chromium-mediated transformation and drug resistance

**DOI:** 10.18632/aging.203917

**Published:** 2022-02-24

**Authors:** Suthakar Ganapathy, Jian Liu, Tianqi Yu, Rui Xiong, Qiang Zhang, Alexandros Makriyannis, Changyan Chen

**Affiliations:** 1 Center for Drug Discovery, Northeastern University, Boston, MA 02115, USA; 2 The Department of Medicine, The First Affiliated Hospital of Nanchang University, Nanchang, Jiangxi, PR China

**Keywords:** Cr(VI), Src, Ras, PKC, Bcl-2, tumorigenesis

## Abstract

Hexavalent chromium [Cr(VI)] pollution is a serious environmental problem, due to not only its toxicity but also carcinogenesis. Although studies reveal several features of Cr(VI)-induced carcinogenesis, the underlying mechanisms of how Cr(VI) orchestrates multiple mitogenic pathways to promote tumor initiation and progression remain not fully understood. Src/Ras and other growth-related pathways are shown to be key players in Cr(VI)-initiated tumor prone actions. The role of protein kinase C (PKC, an important signal transducer) in Cr(VI)-mediated carcinogenesis has not been thoroughly investigated. In this study, using human bronchial/lung epithelial cells and keratinocytes, we demonstrate that PKC activity is increased by transient or chronic Cr(VI) exposure, which plays no role in the activation of Src/Ras signaling and ROS upregulation by this metal toxin. PKC in chronic Cr(VI)-treated cells stabilizes Bcl-2 to mitigate doxorubicin (an anti-cancer drug)-mediated apoptosis. After the suppression of this kinase by GO6976 (a PKC inhibitor), the cells chronically exposed to Cr(VI) partially regain the sensitivity to doxorubicin. However, when co-suppressed PKC and Ras, the chronic Cr(VI)-treated cells become fully responsive to doxorubicin and are unable to be transformed. Taken together, our study provides a new insight into the mechanisms, in which PKC is an indispensable player and cooperates with other mitogenic pathways to achieve Cr(VI)-induced carcinogenesis as well as to establish drug resistance. The data also suggest that active PKC can serve as a potential biomarker for early detection of health damages by Cr(VI) and therapeutic target for developing new treatments for diseases caused by Cr(VI).

## INTRODUCTION

Hexavalent chromium Cr(VI) has long been recognized as one of the environmental metal toxins or carcinogens. Epidemiological studies indicate that drinking water or soil in some industrial areas as well as in many less developing countries are often contaminated with this toxic compound [1, 2]. For example, more than one third of the tested aquifers in California shows a trace of Cr(VI) contamination [3]. Furthermore, during natural geochemical processes or chlorination, water pollution with Cr(VI) is frequently detected in many locations [3–5]. It is proven that a long term contact or inhalation of Cr(VI)-contaminated water, dusts and fumes is closely associated with the onsets of various diseases and malignancies (especially lung or skin cancer) [3–6]. Therefore, environmental exposure to Cr(VI) remains a major public health concern and is considered to pose a high risk for carcinogenesis. One of the mechanisms by which Cr(VI) promotes tumorigenesis is through its aberrant augmenting intracellular ROS (reactive oxygen species) and consequent perturbing genomic stability of cells [7–9]. However, it remains unclear about how Cr(VI) transmits carcinogenic signaling to initiate diseases or cancers. A better understanding of this pollutant will help develop effectively environmental protection to prevent people from Cr(VI) exposure and further from suffering in particular lung or skin cancer.

Cr(VI) has the structural resemblance of phosphate (CrO_4_^−2^-PO_4_^−2^) that can be actively transported into cells and replaces anions (such as phosphates) to disrupt normal cellular activities [3–5]. Frequent inhalation or contact of dusts and fumes contaminated with Cr(VI) is the common form that trigger respiratory or skin diseases, in particular, malignancies thereof. One of the mechanisms of Cr(VI)-induced carcinogenesis is via the induction of oxidative stress, in which TrxR (thioredoxin reductase)/Trx (oxidation of thioredoxin)/Prx (peroxiredoxin) signaling is stimulated, which in turn activates mitogenic-related kinases and further upregulates the expressions of redox-related transcription factors [10, 11]. Trx induced by Cr(VI) is able to perturb Prx function. Since the TrxR/Trx system controls many redox-regulated factors, its abnormality can change a broad spectrum of cellular functions or activities [12, 13]. Although it is not fully understood how Cr(VI) initiates aberrant redox changes in cells, some studies indicate that Cr(VI) reacts with H_2_O_2_ to generate reactive intermediates to influence cell survival, death or inflammation [10–13]. It has also been shown that Cr(VI) preferentially oxidizes certain thiols of proteins that initiates signaling cascade reactions [2, 14, 15]. The structural nature of Cr(VI) permits its passing through the bronchial, skin and gastrointestinal tract. After entering the body, Cr(VI) causes pathological alterations in different tissues or organs.

The Src family members are a group of protein tyrosine kinases and play key roles in initiating growth-related signaling in response to a diverse of stimuli [16–19]. Ras often functions at downstream of Src and exists in two conformations: a GTP-bound active state and GDP-bound inactive state. Ratios of GTP- and GDP-bound Ras are controlled by guanine nucleotide exchange proteins and GTPase-activating proteins, the activity of which responds to activation of extracellular (such as growth factors) or intracellular receptors (like Src) [19–22]. Once interacting with the effectors, signals generated from Ras influence cell growth, migration and other activities. The best characterized effectors of Ras are various serine/threonine kinases, for example, Raf/MAPK, PI3K/Akt or Rho/Ral [23–25]. An aberrant activation of Ras is shown to upregulate ROS and perturb redox state to promote cell transformation or tumorigenesis, including Cr(VI)-induced carcinogenesis [26, 27].

Protein kinase C (PKC) constitutes the canonical signaling pathway downstream of growth-related receptors and functions as a tumor promoter [28–30]. The family of PKC consists of more than 10 isoforms that are serine/threonine kinases [28]. Stimulation of G-protein-coupled or tyrosine kinase receptors (such as Src) activates phospholipase C (PLC) that then triggers transient generations of IP3 and diacylglycerol (DAG). DAG in tern stimulates PKC to elicit phosphorylation cascades, leading to upregulations of genes related genes for promoting cell growth or survival. The activities of the conventional PKC isoforms (such as α and β) depend upon DAG and calcium. Abnormal increases of PKC activity or expression are detected in many types of human malignancies [31–33].

Bcl-2 plays an important role in supporting cell survival or establishing drug resistance in cancer cells [34–36]. Upon apoptotic stimulation, this anti-apoptotic factor translocates from the endoplasmic reticulum (ER) to the mitochondria to prevent cytochrome c releasing [37–39]. In addition, Bcl-2, via its BH1-4 domains, interacts with various pro- or anti-survival factors to regulate apoptosis. It was shown that Bcl-2 bound to Bax (a pro-apoptotic factor) and blocked apoptosis [40]. Post-translational modifications (especially phosphorylation) take part in the regulation of Bcl-2 function [41]. Studies showed that Bcl-2 anti-apoptotic function required the phosphorylation at its serine residue [41, 42]. It was also reported that the augmented level of Bcl-2 phosphorylation correlated well with the increase of the resistance of small cell lung cancer cells to anti-cancer drugs [41, 42]. In addition, PKC was implicated to be involved in Bcl-2 modifications and degradation.

In this study, we used human bronchial epithelia BEAS-2B cells and keratinocytes that are in the first lines of human body in contact with Cr(VI). Our study showed that PKC in these cells was activated by the transient or chronic exposure of this metal toxin, which was in a Src/Ras-independent fashion. The activation of PKC by Cr(VI) was necessary for the cells to undergo transformation as well as to establish the resistance to doxorubicin. These actions appeared involving PKC-induced upregulation of Bcl-2 stability. After the co-inhibition of PKC and Ras, persistent or chronic Cr(VI)-exposed cells could no longer be transformed and regained the sensitivity to doxorubicin. Thus, our study indicates that PKC acts as a critical player in Cr(VI)-mediated carcinogenesis.

## RESULTS

### Cr(VI) stimulates and sustains PKC activity

**Figure 1:**
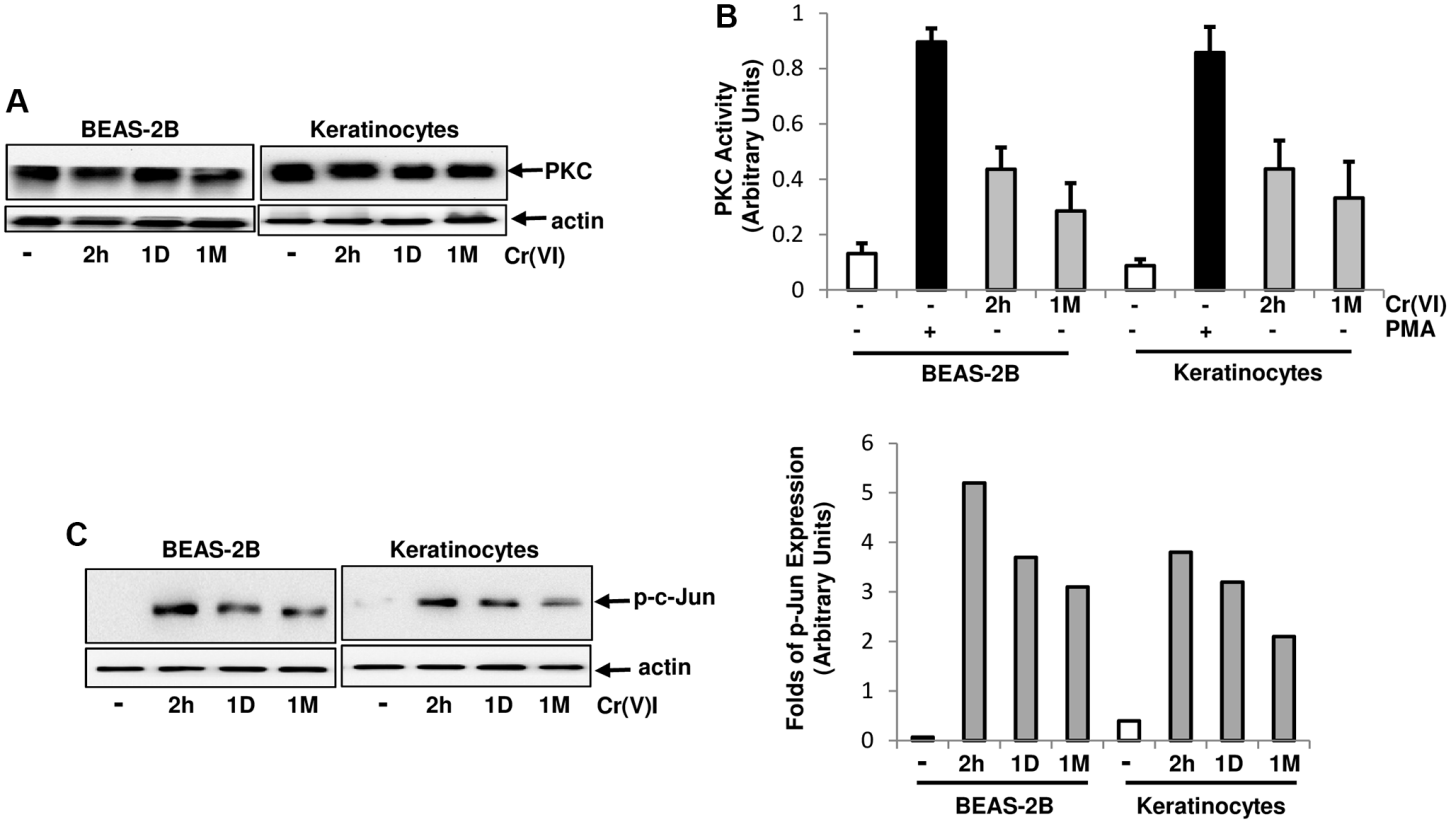
**Activation of PKC by Cr(VI) in BEAS-2B cells and Keratinocytes.** (**A**) Cells were treated with Cr(VI) (2.0 uM) for different times. Subsequently, PKC expression was tested by immunoblotting. Actin was the loading control. (**B**) After Cr(VI) treatment for 2 h or 1 month (1M), PKC activity in the cells was analyzed by PKC kinase activity assay. PMA (0.5 μM) stimulation serves as positive control. The error bars represent standard deviation (SD) (*n* = 5, *p* < 0.01). (**C**) Cells were treated as described above and lysates were prepared. Subsequently, the phosphorylated c-Jun was examined by immunoblotting (left panels). The folds of the proteins induced were also measured and plotted (right panel). Actin was the loading control.

PKC, as an important signal transducer, participates in regulating cell proliferation, migration and apoptosis [28–30]. However, the role of PKC in Cr(VI)-mediated carcinogenesis remains unclear, regardless of the studies showing the involvement of several mitogenic pathways (such as Src/Ras, JNJ/p38) in this metal toxin-induced actions. Therefore, PKC expression in human bronchial epithelial cells and keratinocytes with or without transient (2 h) or chronic exposure (<2 h) to Cr(VI) was first tested by immunoblotting (Figure 1A). The expression levels of this kinase in the cells were not altered by Cr(VI) treatment at different testing times. PKC activity was then analyzed after transient or chronic Cr(VI) exposure (Figure 1B). Phorbol 12-myristate 13-acetate (PMA, a PKC stimulator) was used as a positive control, and strongly stimulated PKC activity. The transient Cr(VI) treatment moderately upregulated PKC activity, which was slightly declined, but sustained in response to the prolonged exposure. AP-1 (a transcription factor) is downstream of PKC and composed of homo- or heterodimers of c-Jun, c-Fos or ATF [43]. To further determine PKC activation, the phosphorylation or activation status of c-Jun in the cells was examined following Cr(VI) exposure at different times (Figure 1C). c-Jun was phosphorylated in the cells received Cr(VI) treatment for 2 h, which was attenuated, but maintained at a moderate level following the chronic exposure (Figure 1C, left panels). The folds of increased, phosphorylated c-Jun were also measured by the phosphor-imager (Figure 1C, right panel).

### PKC activation by Cr(VI) was independent of the Src/Ras signaling

**Figure 2:**
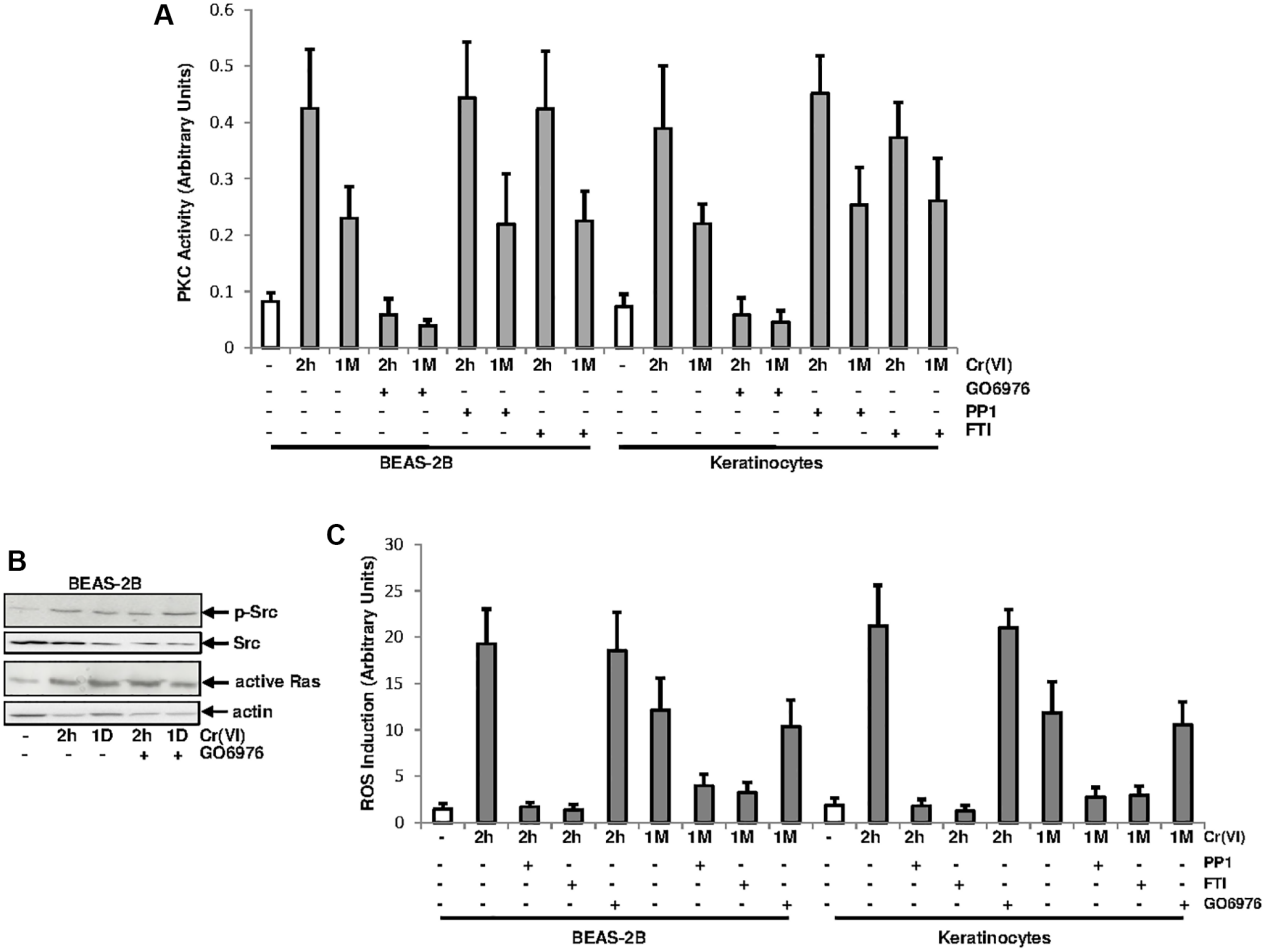
**PKC activation by Cr(VI) was independent of Src/Ras signaling.** (**A**) BEAS-2B cells and keratinocytes were treated with Cr(VI) for 2 h or 1 month in the presence or absence of GO6976 (1.0 μM), FTI (0.25 μM) or PP1 (0.15 μM). Afterwards, lysates were prepared for PKC activity assay. The error bars were SD (*n* = 5, *p* < 0.05). (**B**) BEAS-2B cells were exposed to Cr(VI) for 2 h or 24 h in the presence or absence of GO6976 and then assayed for the expressions of the phosphorylated Src by immunoblotting and active Ras using active Ras pull-down kit. Src and actin were the loading controls. (**C**) Cells were treated as described above and levels of ROS were then analyzed. The error bars were SD (*n* = 5, *p* < 0.01).

Src/Ras signaling pathway was suggested to be cooperated with PKC in regulating different cellular activities by mitogenic stimulation [28–30]. It was not clear about if and how PKC and Src/Ras pathways were interacted in response to Cr(VI) exposure. To test this, PKC activity, after Cr(VI) treatment for different times, was analyzed in the presence and absence of GO6976 (a PKC inhibitor), PP1 [4-amino-5-(4-methyphenyl)-7-(t-butyl) pyrazolo (3.4-d)-pyrimidine, a Src inhibitor] or FTI (farnesyltransferase, a Ras inhibitor) (Figure 2A). GO6976 inhibited the upregulation of PKC induced by transient or chronic Cr(VI) exposure, as expected. However, The addition of PP1 or FTI respectively, played no role in Cr(VI)-mediated PKC upregulation in the cells.

To further determine the involvement of PKC in Cr(VI), via PKC, activated Src or Ras signaling, BEAS-2B cells were treated with Cr(VI) for 2 h or 1 day in the presence or absence of GO6976 and afterwards the activation of Src or Ras was analyzed (Figure 2B). Src was phosphorylated and active Ras was detected in the cells after Cr(VI) exposure at the times tested, suggesting that the inhibition of PKC did not affect Cr(VI)-mediated activation of these two signal transducers.

Cr(VI) was shown to via activating the Src/Ras signaling, aberrantly increased ROS levels and further disrupted redox balance in normal cells [27]. To test the involvement of PKC in Cr(VI)-mediated perturbation of redox signaling, the amounts of ROS was analyzed (Figure 2C). Two hours of Cr(VI) exposure strongly augmented ROS in BEAS-2B cells and keratinocytes, which was slightly reduced, but sustained by the chronic exposure. The addition of PP1 or FTI, but not of GO6976, suppressed Cr(VI)-mediated ROS upregulation. Thus, the results indicate that the increase of ROS by Cr(VI) is regulated by Src/Ras signaling and independent of PKC.

### PKC, via upregulating Bcl-2, desensitizes Cr(VI)-treated cells to doxorubicin-mediated cytotoxicity

**Figure 3:**
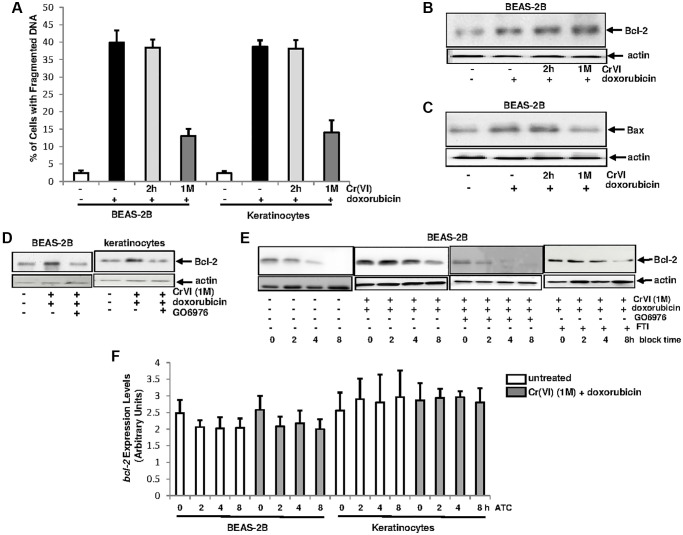
**Upregulation of Bcl-2 by Cr(VI) was via increasing its stability and depends upon PKC.** (**A**) Cells were exposed to Cr(VI) for 2 h and 1 month and their sensitivities to doxorubicin (5 μM)-induced apoptosis were analyzed by DNA fragmentation assay. The error bars were SD (*n* = 5, *p* < 0.01). (**B**) BEAS-2B cells were transiently or persistently exposed to Cr(VI) prior to doxorubicin treatment, and then examined for Bcl-2 expression. Actin was the loading control. (**C**) After the treatments as described above, Bax expression was analyzed. Actin was the loading control. (**D**) After adding GO6976, Bcl-2 expression in the cells received the treatments as described above was tested by immunoblotting. Actin was the loading control. (**E**) Cells chronically exposed to Cr(VI) were treated with doxorubicin or co-treated with GO6976, prior adding CHX (0.5 μM). Bcl-2 expression was then analyzed at different time points of CHX block. Actin was the loading control. (**F**) Cells chronically exposed to Cr(VI) were treated with doxorubicin. Afterwards, actinomycin D (ATC, 1.0 nM) was added and expression levels of *bcl-2* gene were analyzed at different time points of ATC block by real time PCR. The error bars were SD (*n* = 5, *p* < 0.05).

Cr(VI)-initiated cancer cells, via desensitizing the intrinsic cell machinery, often develop drug resistance, which is a major challenge for chemotherapies [43, 44]. In order to explore the mechanisms, the sensitivity of the cells transiently or chronically treated with Cr(VI) to apoptosis induced by doxorubicin (a chemo-drug) was examined (Figure 3A). After being exposed to Cr(VI) for either 2 h or one month, BEAS-2B cells and keratinocytes were then treated with doxorubicin for 48 h and the percentages of the cells with fragmented DNA were measured. Approximately 40% of the cells treated with doxorubicin alone or transiently exposed (2 h) to Cr(VI) underwent apoptosis. In comparison, less than 15% of the cells received chronic Cr(VI) exposure became apoptotic after doxorubicin treatment, indicating that a long-period exposure of Cr(VI) impairs the intrinsic cell death machinery.

Bcl-2 is a pro-survival factor and PKC was shown to be involved in the regulation of Bcl-2 function [41, 42, 45]. To test if and how these two factors might be involved in the resistance to doxorubicin in chronic Cr(VI)-treated cells, the expression of Bcl-2 in Cr(VI)-treated BEAS-2B cells, after doxorubicin treatment, was analyzed by immunoblotting (Figure 3B). A baseline expression of Bcl-2 was detected in untreated cells, which was augmented in the cells treated with doxorubicin alone and co-exposed to transient or chronic Cr(VI). Bax is a pro-apoptotic factor and able to interact with Bcl-2 [46]. The expression of Bax after the same treatments described above was then tested (Figure 3C). The level of Bax in BEAS-2B cells with or without 2 h of Cr(VI) exposure was increased, which did not occur in the cells chronically exposed to Cr(VI). The data indicates that Bax is induced by doxorubicin and chronic Cr(VI) exposure appears hindering Bax induction, which may facilitate in strengthening Bcl-2 anti-apoptotic function.

The involvement of PKC in the promotion of Bcl-2 function during various apoptotic processes has been reported [41–44]. To test the importance of the cooperation of PKC and Bcl-2, BEAS-2B cells and keratinocytes chronically exposed to Cr(VI) were treated with doxorubicin in the presence or absence of GO6976 (Figure 3D). Subsequently immunoblotting for Bcl-2 expression was performed. Again, Bcl-2 expression in BEAS-2B cells or keratinocytes chronically exposed to Cr(VI) was induced by doxorubicin, which was suppressed by GO6976, indicating the linear relationship between these two molecules and also suggesting that this anti-apoptotic factor functions downstream of PKC.

Protein stability is one of the elements for the regulation of protein expressions [47]. Therefore, we tested if the upregulation of Bcl-2 expression by Cr(VI), in response to doxorubicin, was due to the influence of PKC on its degradation process (Figure 3E). The chronic Cr(VI)-exposed BEAS-2B cells were treated with either GO6976 or FTI, prior to adding doxorubicin following blocking protein synthesis by cycloheximide (CHX). Subsequently, Bcl-2 expression levels were examined at different times of CHX block. This anti-apoptotic factor in chronic Cr(VI)-treated cells was much more stable after doxorubicin treatment than that in untreated cells. After the suppression of PKC by GO6976, the kinetics of Bcl-2 degradation in chronic Cr(VI)-treated cells, after doxorubicin treatment, became similar as that of untreated cells. In the presence of FTI, the attenuation of Bcl-2 degradation in the cell co-exposed to chronic Cr(VI) treatment and doxorubicin was unchanged. Next, the stability of *bcl-2* gene in chronic Cr(VI)-treated cells, after the addition doxorubicin, was also examined, using Real-Time PCR analysis (Figure 3F). After the addition of actinomycin D (ATC) to block gene transcription, the levels of *bcl-2* in the treated- or untreated cells were similar, indicating that the increase of Bcl-2 expression by Cr(VI) is not regulated at the transcriptional level. Taken together, the results suggest that PKC, via influencing protein degradation process, stabilizes Bcl-2 in chronic Cr(VI) exposed cells, which is in a Ras independent fashion.

### PKC cooperates with Ras pathway, to promoted Cr(VI)-mediated drug resistance and cell growth

**Figure 4:**
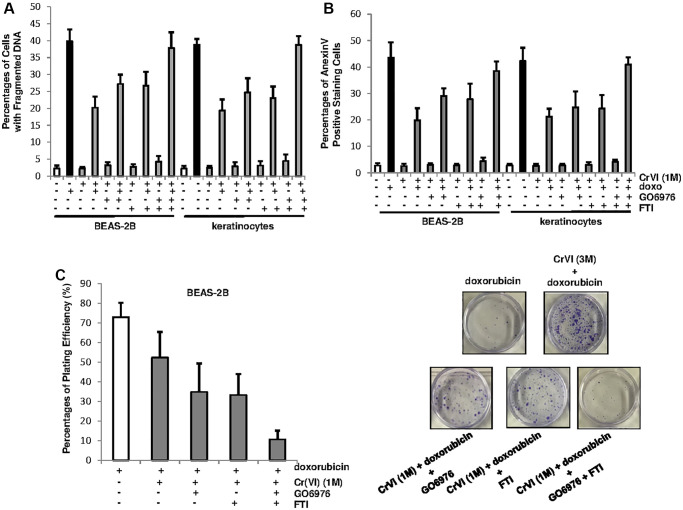
**PKC and Ras cooperate to promote Cr(VI)-mediated drug resistance and long-term survival.** (**A**) Cells with or without chronic Cr(VI) exposure were received different treatments. Subsequently, the percentages of the cells with fragmented DNAs were measured. The error bars were SD (*n* = 5, *p* < 0.01). (**B**) After the same treatments as described above, the cells were subjected to Annexin V apoptotic assay. The error bars were SD (*n* = 5, *p* < 0.01). (**C**) After the treatments described above, colony formation assay was conducted. The plating efficiencies were measured (right panel) and colonies were imaged (right panels). The error bars were SD (*n* = 5, *p* < 0.05).

To further test the role of PKC or Ras in our experimental setting, BEAS-2B cells and keratinocytes, after being chronically exposed to Cr(VI), were treated with doxorubicin in the presence or absence of GO6976, FTI or both for 48 h and then subjected DNA fragmentation analysis (Figure 4A). The cells chronically exposed to Cr(VI) were much less sensitive to doxorubicin for the induction of apoptosis than the control cells. The inhibition of PKC or Ras, respectively, partially restored the sensitivity of chronic Cr(VI) treated cells (about 25%) to doxorubicin for the induction of apoptosis. When being co-treated with GO6976 and FTI, more than 40% of the cells chronically exposed to Cr(VI) were sensitized to doxorubicin-induced apoptosis. The similar results were obtained from Annexin V analysis (Figure 4B). The results suggest that PKC and Ras both take part in the regulation of Cr(VI)-mediated drug resistance, but function in the separate, but cooperative pathways.

To further test the effect of PKC or Ras on long-term survival of chronic Cr(VI)-exposed cells in response to doxorubicin treatment, the clonogenicity assay was conducted (Figure 4C). BEAS-2B cells chronically exposed to Cr(VI) were treated with doxorubicin in the presence or absence of GO6976 or FTI and then grew for 15 days to allow forming colonies. The plating efficiencies showed that chronic Cr(VI) exposure enhanced colony formation capability of the cells in response to doxorubicin (Figure 4C, left panel). The inhibition of PKC or Ras respectively, partially reduced the ability of chronic Cr(VI)-treated cells to doxorubicin. In comparison, the co-suppression of these two pathways abolished the resistance to this chemo drug. The images of the colonies clearly indicated the effect of the suppression of PKC, Ras or both on the clonogenicity of the cells chronically exposed to Cr(VI), following doxorubicin treatment (Figure 4C, right panels). Thus, the data indicate that persistent Cr(VI) exposure, via activating PKC and Ras, not only promotes cell survival, but also drug resistance.

### PKC and Ras are required for Cr(VI)-mediated transformation

**Figure 5:**
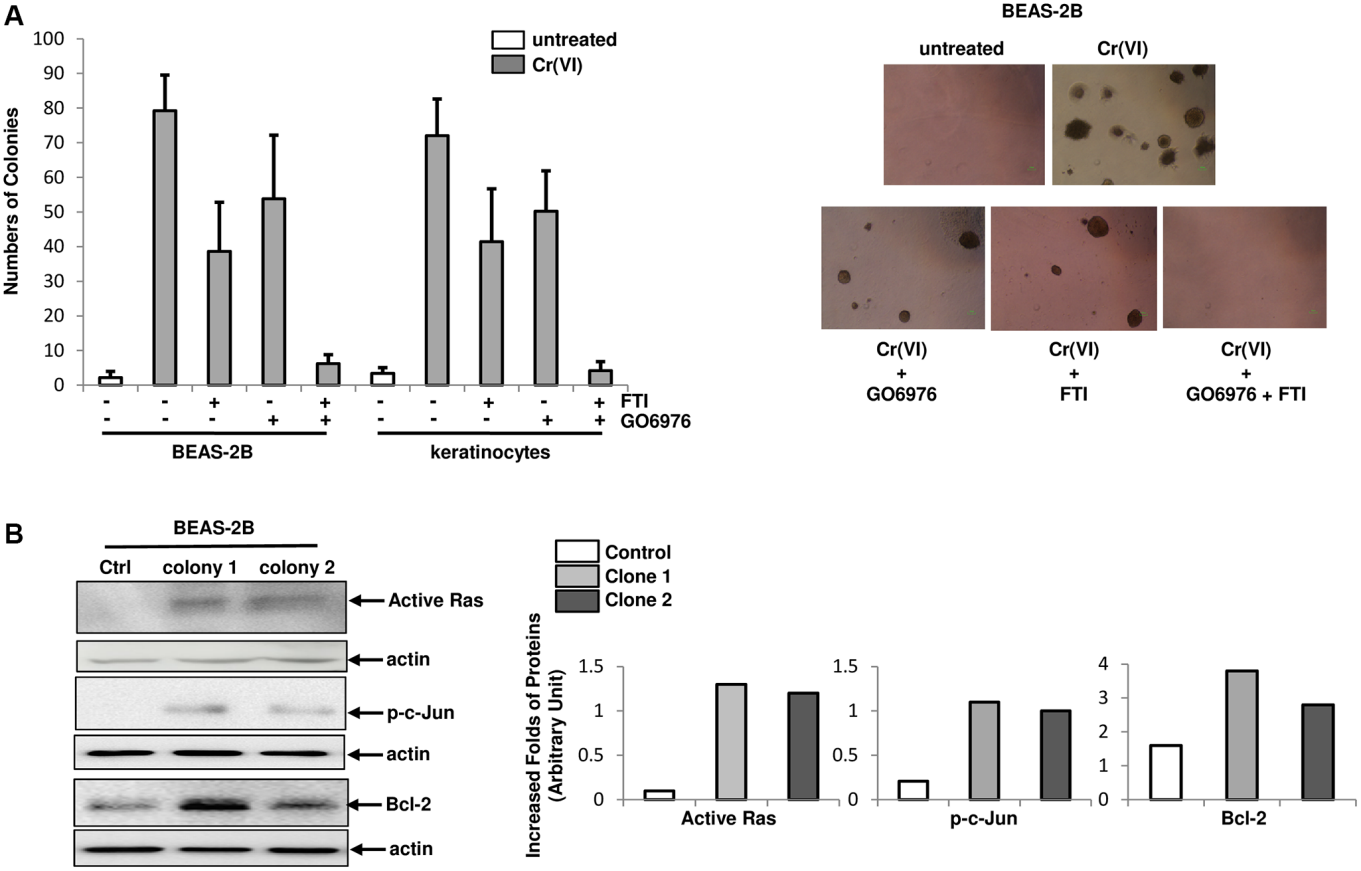
**Role of PKC or Src/Ras signaling in Cr(VI)-mediated transformation.** (**A**) Cells were cultured in soft agar containing Cr(VI) or Cr(VI) plus PP1, FTI or GO6976, respectively for another 4.5 months. Untreated cells grown in soft agar medium served as the control. The numbers of the colonies were counted (left panel) and examples of colonies from the treatments were imaged (right panels). The error bars were SD (*n* = 5, *p* < 0.05). (**B**) Expression of active Ras, phosphorylated c-Jun and Bcl-2 in two Cr(VI) transformed colonies were examined by immunoblotting (left panels). The folds of the proteins induced were measured and then plotted (right panels). Actin was the loading control.

To test the effect of chronic Cr(VI) exposure on cellular transformation, BEAS-2B cells and keratinocytes were grown in soft agar medium containing Cr(VI) in the presence or absence of FTI, GO6976 or both for 4.5 months and the numbers of colonies were then counted (Figure 5A). A few of untreated control BEAS-2B cells or keratinocytes were formed colonies (Figure 5A, left panel). In comparison, approximately 5% of chronic Cr(VI)-treated cells formed colonies in soft agar medium. The co-treatment of Cr(VI) with FTI or GO6976, respectively, partially suppressed this transformation process. In the presence of both GO6976 and FTI, this metal toxin became unable to transform the cells. The images showed the colonies from BEAS-2B cells received different treatments (Figure 5A, right panels). Furthermore, the expressions of active Ras, phosphorylated c-Jun or Bcl-2 in two chronic Cr(VI)-treated, transformed colonies were analyzed, using active Ras pull down assay or immunoblotting analysis (Figure 5B, left panels) and folds of the inductions of these factors were measured (Figure 5B, right panels). Consistently, active Ras, p-c-Jun and increased Bcl-2 were detected in Cr(VI)-transformed BEAS-2B cells. The results further support the notion that Ras and PKC signaling pathways are indispensable elements in Cr(VI)-mediated transformation.

## DISCUSSION

Hexavalent chromium VI [Cr(VI)] is a well-known environmental hazard that causes various human diseases, including cancer [1–3]. Studies revealed that the exposure to this metal toxin is able to disrupt intracellular redox balance for promoting tumorigenesis [3]. Previously, we demonstrated that persistent or chronic Cr(VI) exposure, via activating Src/Ras signaling axis, aberrantly upregulated intracellular ROS and further caused normal cells to undergo malignant transformation [27]. Because environmental Cr(VI) pollution is mainly related to its chronic exposure, the effect of which is the focus of our current investigation. We showed that transient Cr(VI) exposure activated PKC in human lung epithelial BEAS-2B cells or keratinocytes, which was sustained by its prolonged treatment. PKC activation under such conditions was independent of Src/Ras signaling, but played no role in the perturbation of redox states. Furthermore, Cr(VI) exposure, via attenuating Bcl-2 degradation, increased its expression and function, which promoted cell survival as well as their resistance to doxorubicin-induced apoptosis, in a PKC dependent fashion. However, Cr(VI) required the cooperation of PKC and Src/Ras pathways to achieve a full transformational process.

Epidemiological studies indicate that exposure to Cr(VI) contaminated soil, water or in some industrial areas is often associated with a high incidence of various diseases (such as anemia or asthma) and tumors (especially lung or skin malignancies) [3–5]. After being inhaled or absorbed into the body, Cr(VI) rapidly enters cells through anion transporters in the plasma membrane [12–14]. Afterwards, Cr(VI) is able to disrupt redox balance and initiate or promote tumorigenesis. The family of Src members (such as Fyn or Src) could transmit signals induced by this metal toxin, and further activate Ras [16–19]. The functional domains of Src kinase contain an amino-terminal myristoylation sequence for membrane targeting. The amino-terminal region of Src is also responsible for specific interactions with downstream effectors or parallel kinases, which then trigger phosphorylation chain reactions. Src functions as an intracellular receptor and connects plasma membrane signals to downstream effectors for eliciting various cellular activities. Efforts have been made for elucidating the mechanisms by which Cr(VI) mobilizes growth-related signaling in cells. G-proteins, Ras, mitogen-activated kinase (MAPK) are important factors in the regulation of various cellular functions. Here, we demonstrated that Src/Ras signaling was activated by transient and persistent Cr(VI) exposures in human lung epithelial cells or keratinocytes. Because Src family members are involved in mediating receptor signals triggered by cytokines, antigens or extracellular matrix receptors, we in this study further proved the role of Src/Ras signaling pathway in initiating Cr(VI)-mediated carcinogenesis or transformation.

The introduction of mutant *RAS* into mammalian cells alters physiological cellular responses, and often initiates human diseases. Aberrant, hyperactive Ras is a pivotal element in initiating tumorigenesis upon Cr(VI) exposure [27]. By binding to multiple downstream effectors, Ras activates distinct signaling pathways to regulate various cellular activities [23–25]. It was shown that Cr(VI)-induced activation of ERK1/2 (Ras downstream effectors) was different from those activated by mitogens [27, 48, 49]. By mobilizing Ras/ERK1/2 signaling, Cr(VI) exposure upregulates intracellular free radicles and in turn disrupts redox homeostasis, leading to oxidative stress and subsequent carcinogenesis. In contrast, the transcriptional machinery activated by mitogenic signaling often stimulates expressions of growth-related genes. The present study again proves that Cr(VI), by utilizing Src/Ras signaling axis, aberrantly increases ROS, which contributes greatly to Cr(VI)-induced carcinogenesis.

Stimulation of G-protein-coupled and tyrosine kinase receptors (such as Src) activate phospholipase C (PLC), which then causes the generations of IP3 and diacylglycerol (DAG). These two PLC products in turn activate several intracellular kinases, including PKC [28, 29]. Increases of PKC activities, especially the conventional PKC isoforms (α and β), have been reported to be associated with the development of various types of cancers [28–30]. Using PKC inhibitor GO6976 that mainly suppresses PKC α and β isoforms, we demonstrated that Cr(VI), through activating PKC, upregulates anti-apoptotic factor Bcl-2 in response doxorubicin-mediated cytotoxic stimulation. In this process, Cr(VI), by interfering with Bcl-2 protein degradation process, increases its stability. The underlying mechanisms of how Cr(VI) interferes with the processes of ubiquitination and degradation remain to be further investigated. Importantly, because the existence of multiple isoforms of PKC with their redundant or overlapping functions, the suppression of PKC by its inhibitors (such as GO6076) appears less toxic. Therefore, our study indicates that PKC inhibitor-based drugs or compounds may be potential candidates for developing new strategies to treat diseases or tumors caused by Cr(VI) or for designing new reagents antagonizing Cr(VI) pollution.

An increase of Bcl-2 activity or expression in cells is closely associated with augmenting tumorigenicity or drug resistance [34–36]. Protein phosphorylation and ubiquitination are important post-translational modification processes in the regulation of Bcl-2 pro-survival activity. PKC was suggested to be one of the kinases responsible for Bcl-2 phosphorylation and further upregulating its function [46–48]. In this study, we demonstrated that PKC in chronic Cr(VI)-treated cells influences Bcl-2 by mitigating its degradation to antagonize doxorubicin-induced cytotoxicity. The cooperation of PKC and Bcl-2 by Cr(VI) in our experimental setting is independent of ROS, which appears contradictory to other’s observation that Bcl-2 degradation during oxidative stress is influenced by free radicles [50]. It is possible that different types or different amounts of reactive species are generated by Cr(VI) exposure, which was unable to affect Bcl-2 expression or function. In addition, Bax upregulation was impaired in the cells chronically exposed to Cr(VI) after being challenged by doxorubicin. Therefore, the resistance of these cells to doxorubicin also in part contributes to the disruption of this pro-apoptotic factor, the mechanisms of which is under way for the investigation. Overall, our study indicates that Bcl-2 upregulation observed here is an important factor in strengthening and sustaining Cr(VI)-mediated drug resistance and transformational action.

In summary, our study demonstrate the mechanism, i.e., the differential activation of different signaling pathways by Cr(VI) exposure initiates and promotes transformation in human lung epithelial BEAS-2B cells and keratinocytes. Using the inhibitors to dissect mitogenic signal pathways, we identified at least two pathways: Src/Ras and PKC are involved in Cr(VI)-induced malignant transformation. The combination of the upregulation of ROS by activated Src/Ras signaling and of Bcl-2 by PKC accounts for Cr(VI)-mediated tumor promotion. Our investigation provides an insight into the mechanism of how PKC acts as an indispensable element in maintaining Cr(VI)-mediated carcinogenic pressure as well as for establishing drug resistance. In addition, this study identifies the targets that can be used for developing new strategies for protecting the environment from Cr(VI) pollution and new therapeutic approaches for treating diseases or malignancies caused by Cr(VI) exposure.

## MATERIALS AND METHODS

### Cells and reagents

Human BEAS-2B lung epithelial cells and human keratinocytes were purchased from ATCC (Manassas, VA). The cells grew in RPMI 1640 supplemented with 10% heat-inactivated fetal bovine serum (Invitrogen, CA) in a 5% CO_2_ humidified atmosphere at 37°C. The authentication of these cell lines is based on the information provided by the company. In addition, the cells were periodically authenticated by monitoring cell morphology and growth curves. The concentration of Cr(VI) used in this study is (2.0 μM). The transient Cr(VI) exposure represent the treatment for 2 h and more than 2 h of the exposures are stated in the text as the persistent or chronic treatment.

Cr(VI), GO6976, FTI, PP1, PMA and doxorubicin were purchased from Sigman (St Louis, MO, USA). Antibodies against p-Src, Src, β-actin, pan-PKC, Bax, p-c-Jun and c-Jun were purchased from Santa Cruz Biotechnology (Santa Cruz, CA, USA). Anti-Bcl-2 antibody was from Cell Signaling Technology (Danvers, MA, USA). The authentication information was provided by each company.

### Immunoblotting analysis

After treatments, cells were harvested, washed with 1 x PBS twice and lysed in the lysis buffer (50 mM Tris-HCl, pH 8.0, 150 mM NaCl, 1% Triton-X114, 0.5% sodium deoxycholate, 0.1% sodium dodecyl sulfate, supplemented with protease and phosphatase inhibitor cocktails (Thermo Scientific, Waltham, MA, USA). Subsequently, cell lysates were separated by 10% SDS-PAGE gels (Thermo Scientific, Waltham, MA, USA) and transferred to Immobilon-P membrane (Millipore, Burlington, MA, USA). After blocked with TBS containing 5% of dried milk, membranes were probed with primary antibodies, followed by added ECL, anti-rabbit IgG, anti-mouse IgG or anti-goat IgG secondary antibody (1:5,000-10,000) and then imaged. All immunoblotting experiments were conducted at least twice.

### PKC activity assay

PKC activity was tested using the kit from Abcam (Cambridge, MA, USA). According to the protocol provided by the company: after treatments, samples were added into each well of a 96 well-plate and then suspended with 10 μl of diluted ATP per well. After keeping at 30°C for 90 min on a rotating shaker, 40 μl of the antibody against corresponding phosphor-specific substrate was added into each well. Following 60 min of incubation, liquid in each well was aspirated and samples were washed with the wash buffer. Subsequently, a second antibody was added followed by the substrate. Finally, the stop solution was added and absorbance was read by a microplate reader. For each treatment, 5 wells were used and the experiments were repeated twice.

### Active Ras pull-down assay

After treatments, cell lysates were collected and assayed by the active Ras pull-down and detection kit, as instructed by the manufacturer (Thermo Scientific, Waltham, MA, USA). Briefly, the GTP-form of Ras was pulled down by a GST-fusion protein with the Ras-binding domain (RBD) of Raf attached to glutathione agarose. The pull-down complexes were washed and separated on a 10% SDS-PAGE gel and immunoblotted with an anti-pan-Ras antibody. For loading controls, equal amounts of lysates as that for the assay were subjected to immunoblotting and then probed with anti- β-actin antibody. The assay was conducted at least two times.

### ROS analysis

After treatments, cells were washed with ice-cold PBS and resuspended in 5 μg/ml of 2′, 7′-dichlorodihydrofluorescein diacetate (DCF) (Thermo Fisher Scientific, MA, USA). Following by incubation for 10 min at room temperature, the samples were immediately analyzed.

### DNA fragmentation apoptotic assay

A flow cytometric analysis was performed using a FACScan (BD Biosciences, Franklin Lakes, NJ, USA). The data analysis was performed using the Cell-Fit software program (BD Biosciences). Cell-Fit receives data from the flow cytometer and provides real-time statistical analysis, computed at 1-s intervals, and also discriminates doublets or adjacent particles. Cells with sub-G_0_-G_1_ DNA contents after staining with propidium iodide were counted as apoptotic cells. In brief, after treatments, the cells were harvested and then fixed in 70% cold ethanol. Afterward, cells were stained with 0.01 μg/ml of propidium iodide containing 1.5 ng/ml of RNase. DNA contents of cells were then tested using a FACScan machine. The experiments were repeated three times to verify reproducibility.

### Clonogenicity assay

Clonogenicity assay is to measure the ability of a cell to grow and form a colony. Cells (1000 cells/dish) with or without chronic Cr(VI) exposure were seeded into 10 cm diameter plates and allowed to adhere overnight. Cells then were treated with doxorubicin in the presence or absence of different inhibitors and grown for 15 days meanwhile the culture medium was changed every 3 days. Afterwards, cells were fixed with the fixation solution containing 1% methanol and 1% formaldehyde, and stained with 0.5% crystal violet. A cluster containing more than 60 cells was considered as a colony. The numbers of colonies were counted using an optical microscope and plating efficiencies were plotted as percentages of numbers of treated cells versus untreated cells. For each treatment, 5 dishes were used.

### Soft agar assay

Cells (2500 cells/dish) were grown in soft agar medium in the presence or absence of Cr(VI) or Cr(VI) plus the inhibitors for 4.5 months. Meanwhile, 0.5 ml of fresh medium with or without Cr(VI) or Cr(VI) plus the inhibitors was added on the top of the soft agar every 4 days. At the end of experiments, plates were stained with crystal violet and colonies were counted under a dissecting microscope.

### Statistical analysis

Statistical analysis was performed using a two-tailed Student’s *t* test for comparison of two groups or a one-way ANOVA analysis. Standard deviations are displayed in the figures. A *p* value < 0.05 was considered significant.

## References

[1] Fagliano JA, Savrin J, Udasin I, Gochfeld M. Community exposure and medical screening near chromium waste sites in New Jersey. Regul Toxicol Pharmacol. 1997; 26:S13–22. 10.1006/rtph.1997.1134 9380833

[2] Myers CR. The effects of chromium(VI) on the thioredoxin system: implications for redox regulation. Free Radic Biol Med. 2012; 52:2091–107. 10.1016/j.freeradbiomed.2012.03.013 22542445 PMC3955998

[3] Costa M. Toxicity and carcinogenicity of Cr(VI) in animal models and humans. Crit Rev Toxicol. 1997; 27:431–42. 10.3109/10408449709078442 9347224

[4] Liu K, Husler J, Ye J, Leonard SS, Cutler D, Chen F, Wang S, Zhang Z, Ding M, Wang L, Shi X. On the mechanism of Cr (VI)-induced carcinogenesis: dose dependence of uptake and cellular responses. Mol Cell Biochem. 2001; 222:221–9. 10.1023/A:1017938918686 11678606

[5] Zhitkovich A. Chromium in drinking water: sources, metabolism, and cancer risks. Chem Res Toxicol. 2011; 24:1617–29. 10.1021/tx200251t 21766833 PMC3196244

[6] Chromium (VI); CASRN 18540-29-9. Washington, DC: Environmental Protection Agency. 1999; https://cfpub.epa.gov/ncea/iris/iris_documents/documents/subst/0144_summary.pdf.

[7] Shi XL, Dalal NS. One-electron reduction of chromate by NADPH-dependent glutathione reductase. J Inorg Biochem. 1990; 40:1–12. 10.1016/0162-0134(90)80034-u 2178178

[8] Suzuki Y, Fukuda K. Reduction of hexavalent chromium by ascorbic acid and glutathione with special reference to the rat lung. Arch Toxicol. 1990; 64:169–76. 10.1007/BF02010721 2372230

[9] Shi X, Dong Z, Dalal NS, Gannett PM. Chromate-mediated free radical generation from cysteine, penicillamine, hydrogen peroxide, and lipid hydroperoxides. Biochim Biophys Acta. 1994; 1226:65–72. 10.1016/0925-4439(94)90060-4 8155741

[10] Chuang SM, Liou GY, Yang JL. Activation of JNK, p38 and ERK mitogen-activated protein kinases by chromium(VI) is mediated through oxidative stress but does not affect cytotoxicity. Carcinogenesis. 2000; 21:1491–500. 10.1093/carcin/21.8.1491 10910949

[11] Tessier DM, Pascal LE. Activation of MAP kinases by hexavalent chromium, manganese and nickel in human lung epithelial cells. Toxicol Lett. 2006; 167:114–21. 10.1016/j.toxlet.2006.08.015 17045426

[12] Wood ZA, Poole LB, Karplus PA. Peroxiredoxin evolution and the regulation of hydrogen peroxide signaling. Science. 2003; 300:650–3. 10.1126/science.1080405 12714747

[13] Wood ZA, Schröder E, Robin Harris J, Poole LB. Structure, mechanism and regulation of peroxiredoxins. Trends Biochem Sci. 2003; 28:32–40. 10.1016/s0968-0004(02)00003-8 12517450

[14] Peskin AV, Low FM, Paton LN, Maghzal GJ, Hampton MB, Winterbourn CC. The high reactivity of peroxiredoxin 2 with H(2)O(2) is not reflected in its reaction with other oxidants and thiol reagents. J Biol Chem. 2007; 282:11885–92. 10.1074/jbc.m700339200 17329258

[15] Chromium in drinking water. WHO/SDE/WSH. 2004; https://www.who.int/water_sanitation_health/publications/chromium/en/.

[16] Courtneidge SA, Fumagalli S, Koegl M, Superti-Furga G, Twamley-Stein GM. The Src family of protein tyrosine kinases: regulation and functions. Development. 1993 (Suppl); 119:57–64. 8049488

[17] Elias D, Ditzel HJ. The potential of Src inhibitors. Aging (Albany NY). 2015; 7:734–5. 10.18632/aging.100821 26454527 PMC4637194

[18] Irby RB, Yeatman TJ. Role of Src expression and activation in human cancer. Oncogene. 2000; 19:5636–42. 10.1038/sj.onc.1203912 11114744

[19] Ishizawar R, Parsons SJ. c-Src and cooperating partners in human cancer. Cancer Cell. 2004; 6:209–14. 10.1016/j.ccr.2004.09.001 15380511

[20] Lowy DR, Willumsen BM. Function and regulation of ras. Annu Rev Biochem. 1993; 62:851–91. 10.1146/annurev.bi.62.070193.004223 8352603

[21] Mattos C. Ras: structural details to guide direct targeting. Aging (Albany NY). 2015; 7:344–5. 10.18632/aging.100754 26080945 PMC4505154

[22] Yang JJ, Kang JS, Krauss RS. Ras signals to the cell cycle machinery via multiple pathways to induce anchorage-independent growth. Mol Cell Biol. 1998; 18:2586–95. 10.1128/MCB.18.5.2586 9566878 PMC110638

[23] Feig LA, Urano T, Cantor S. Evidence for a Ras/Ral signaling cascade. Trends Biochem Sci. 1996; 21:438–41. 10.1016/s0968-0004(96)10058-x 8987400

[24] Marte BM, Downward J. PKB/Akt: connecting phosphoinositide 3-kinase to cell survival and beyond. Trends Biochem Sci. 1997; 22:355–8. 10.1016/s0968-0004(97)01097-9 9301337

[25] Hughes PE, Oertli B, Han J, Ginsberg MH. R-Ras regulation of integrin function. Methods Enzymol. 2001; 333:163–71. 10.1016/s0076-6879(01)33054-9 11400334

[26] Zamkova M, Khromova N, Kopnin BP, Kopnin P. Ras-induced ROS upregulation affecting cell proliferation is connected with cell type-specific alterations of HSF1/SESN3/p21Cip1/WAF1 pathways. Cell Cycle. 2013; 12:826–36. 10.4161/cc.23723 23388456 PMC3610730

[27] Ganapathy S, Li P, Lafontant J, Xiong R, Yu T, Zhang G, Chen C. Chromium IV exposure, via Src/Ras signaling, promotes cell transformation. Mol Carcinog. 2017; 56:1808–15. 10.1002/mc.22639 28218450

[28] Griner EM, Kazanietz MG. Protein kinase C and other diacylglycerol effectors in cancer. Nat Rev Cancer. 2007; 7:281–94. 10.1038/nrc2110 17384583

[29] Newton AC. Protein kinase C: perfectly balanced. Crit Rev Biochem Mol Biol. 2018; 53:208–30. 10.1080/10409238.2018.1442408 29513138 PMC5901981

[30] Isakov N. Protein kinase C (PKC) isoforms in cancer, tumor promotion and tumor suppression. Semin Cancer Biol. 2018; 48:36–52. 10.1016/j.semcancer.2017.04.012 28571764

[31] Blobe GC, Obeid LM, Hannun YA. Regulation of protein kinase C and role in cancer biology. Cancer Metastasis Rev. 1994; 13:411–31. 10.1007/BF00666107 7712599

[32] Koivunen J, Aaltonen V, Peltonen J. Protein kinase C (PKC) family in cancer progression. Cancer Lett. 2006; 235:1–10. 10.1016/j.canlet.2005.03.033 15907369

[33] Garg R, Benedetti LG, Abera MB, Wang H, Abba M, Kazanietz MG. Protein kinase C and cancer: what we know and what we do not. Oncogene. 2014; 33:5225–37. 10.1038/onc.2013.524 24336328 PMC4435965

[34] Vervloessem T, La Rovere R, Bultynck G. Antagonizing Bcl-2's BH4 domain in cancer. Aging (Albany NY). 2015; 7:748–9. 10.18632/aging.100828 26525307 PMC4637201

[35] Yang E, Korsmeyer SJ. Molecular thanatopsis: a discourse on the BCL2 family and cell death. Blood. 1996; 88:386–401. 10.1182/blood.V88.2.386.bloodjournal882386 8695785

[36] Lee S, Chari NS, Kim HW, Wang X, Roop DR, Cho SH, DiGiovanni J, McDonnell TJ. Cooperation of Ha-ras and Bcl-2 during multistep skin carcinogenesis. Mol Carcinog. 2007; 46:949–57. 10.1002/mc.20334 17538944

[37] Zamzami N, Brenner C, Marzo I, Susin SA, Kroemer G. Subcellular and submitochondrial mode of action of Bcl-2-like oncoproteins. Oncogene. 1998; 16:2265–82. 10.1038/sj.onc.1201989 9619836

[38] Luo X, Budihardjo I, Zou H, Slaughter C, Wang X. Bid, a Bcl2 interacting protein, mediates cytochrome c release from mitochondria in response to activation of cell surface death receptors. Cell. 1998; 94:481–90. 10.1016/s0092-8674(00)81589-5 9727491

[39] Green DR. Apoptotic pathways: the roads to ruin. Cell. 1998; 94:695–8. 10.1016/s0092-8674(00)81728-6 9753316

[40] Danial NN. BCL-2 family proteins: critical checkpoints of apoptotic cell death. Clin Cancer Res. 2007; 13:7254–63. 10.1158/1078-0432.CCR-07-1598 18094405

[41] Ito T, Deng X, Carr B, May WS. Bcl-2 phosphorylation required for anti-apoptosis function. J Biol Chem. 1997; 272:11671–3. 10.1074/jbc.272.18.11671 9115213

[42] Breitschopf K, Haendeler J, Malchow P, Zeiher AM, Dimmeler S. Posttranslational modification of Bcl-2 facilitates its proteasome-dependent degradation: molecular characterization of the involved signaling pathway. Mol Cell Biol. 2000; 20:1886–96. 10.1128/MCB.20.5.1886-1896.2000 10669763 PMC85374

[43] Pritchard DE, Ceryak S, Ramsey KE, O’Brien TJ, Ha L, Fornsaglio JL, Stephan DA, Patierno SR. Resistance to apoptosis, increased growth potential, and altered gene expression in cells that survived genotoxic hexavalent chromium [Cr(VI)] exposure. Mol Cell Biochem. 2005; 279:169–81. 10.1007/s11010-005-8292-2 16283527 PMC2080352

[44] Nickens KP, Patierno SR, Ceryak S. Chromium genotoxicity: A double-edged sword. Chem Biol Interact. 2010; 188:276–88. 10.1016/j.cbi.2010.04.018 20430016 PMC2942955

[45] Kappelmann M, Bosserhoff A, Kuphal S. AP-1/c-Jun transcription factors: regulation and function in malignant melanoma. Eur J Cell Biol. 2014; 93:76–81. 10.1016/j.ejcb.2013.10.003 24315690

[46] Fletcher JI, Meusburger S, Hawkins CJ, Riglar DT, Lee EF, Fairlie WD, Huang DC, Adams JM. Apoptosis is triggered when prosurvival Bcl-2 proteins cannot restrain Bax. Proc Natl Acad Sci U S A. 2008; 105:18081–7. 10.1073/pnas.0808691105 18981409 PMC2577705

[47] Hicke L. Protein regulation by monoubiquitin. Nat Rev Mol Cell Biol. 2001; 2:195–201. 10.1038/35056583 11265249

[48] Thompson CM, Proctor DM, Suh M, Haws LC, Hébert CD, Mann JF, Shertzer HG, Hixon JG, Harris MA. Comparison of the effects of hexavalent chromium in the alimentary canal of F344 rats and B6C3F1 mice following exposure in drinking water: implications for carcinogenic modes of action. Toxicol Sci. 2012; 125:79–90. 10.1093/toxsci/kfr280 22011396 PMC3243750

[49] Thompson CM, Proctor DM, Haws LC, Hébert CD, Grimes SD, Shertzer HG, Kopec AK, Hixon JG, Zacharewski TR, Harris MA. Investigation of the mode of action underlying the tumorigenic response induced in B6C3F1 mice exposed orally to hexavalent chromium. Toxicol Sci. 2011; 123:58–70. 10.1093/toxsci/kfr164 21712504 PMC3164443

[50] Liu YB, Gao X, Deeb D, Arbab AS, Gautam SC. Pristimerin Induces Apoptosis in Prostate Cancer Cells by Down-regulating Bcl-2 through ROS-dependent Ubiquitin-proteasomal Degradation Pathway. J Carcinog Mutagen. 2013 (Suppl 6); 005. 10.4172/2157-2518.S6-005 24877026 PMC4035051

